# Efficient production and secretion of bovine β-lactoglobulin by *Lactobacillus casei*

**DOI:** 10.1186/1475-2859-6-12

**Published:** 2007-04-06

**Authors:** Stéphane Hazebrouck, Laetitia Pothelune, Vasco Azevedo, Gérard Corthier, Jean-Michel Wal, Philippe Langella

**Affiliations:** 1 INRA, Unité d'Immuno-Allergie Alimentaire, CEA de Saclay, 91191 Gif-sur-Yvette, France; 2 INRA, Unité d'Ecologie et Physiologie du Système Digestif, 78352 Jouy-en-Josas cedex, France; 3 Instituto de Ciências Biológicas, Universidade Federal de Minas Gerais, Belo Horizonte – MG, Brasil

## Abstract

**Background:**

Lactic acid bacteria (LAB) are attractive tools to deliver therapeutic molecules at the mucosal level. The model LAB *Lactococcus lactis *has been intensively used to produce and deliver such heterologous proteins. However, compared to recombinant lactococci, lactobacilli offer some advantages such as better survival in the digestive tract and immunomodulatory properties. Here, we compared different strategies to optimize the production of bovine β-lactoglobulin (BLG), a major cow's milk allergen, in the probiotic strain *Lactobacillus casei *BL23.

**Results:**

Using a nisin-inducible plasmid system, we first showed that *L. casei *BL23 strain could efficiently secrete a reporter protein, the staphylococcal nuclease (Nuc), with the lactococcal signal peptide SP_Usp45 _fused to its N-terminus. The fusion of SP_Usp45 _failed to drive BLG secretion but led to a 10-fold increase of intracellular BLG production. Secretion was significantly improved when the synthetic propeptide LEISSTCDA (hereafter called LEISS) was added to the N-terminus of the mature moiety of BLG. Secretion rate of LEISS-BLG was 6-fold higher than that of BLG alone while intracellular production reached then about 1 mg/L of culture. The highest yield of secretion was obtained by using Nuc as carrier protein. Insertion of Nuc between LEISS and BLG resulted in a 20-fold increase in BLG secretion, up to 27 μg/L of culture. Furthermore, the lactococcal *nisRK *regulatory genes were integrated into the BL23 chromosome. The *nisRK *insertion allowed a decrease of BLG synthesis in uninduced cultures while BLG production increased by 50% after nisin induction. Moreover, modification of the induction protocol led to increase the proportion of soluble BLG to around 74% of the total BLG production.

**Conclusion:**

BLG production and secretion in *L. casei *were significantly improved by fusions to a propeptide enhancer and a carrier protein. The resulting recombinant strains will be further tested for their ability to modulate the immune response against BLG via mucosal delivery in a cow's milk allergy model in mice.

## Background

Lactic acid bacteria are non-invasive and non-pathogenic Gram-positive bacteria with GRAS (generally regarded as safe) status that are widely used for food-processing and preservation. In addition, some strains were reported to exert probiotic effects [1-5]. Using the Nisin-Controlled Expression (NICE) system, β-lactoglobulin (BLG), a major cow's milk allergen, was successfully produced in *Lactococcus lactis *[6-8]. Administrations of BLG-producing lactococci to mice has been shown to induce a mucosal immune response that could partially prevent mice from a further sensitization to BLG [6,9]. However, *L. lactis *is rapidly lysed in each compartment of the digestive tract [10] whereas other LAB, such as lactobacilli, exhibit a greater resistance to the gastric environment and a better survival. Moreover, recent works suggest that some lactobacilli have stronger adjuvant properties than *L. lactis *[11]. Lactobacilli may thus appear as more attractive candidates to deliver therapeutic proteins to the intestinal mucosa. Unfortunately, studies with recombinant lactobacilli are often impaired by the lower levels of antigen production compared to those obtained with *L. lactis *[12]. This is a major concern since mucosal immune response depends on the amount of antigen delivered by the bacterial vector [13].

We previously described the construction of *L. casei *strains carrying a chromosomal BLG expression cassette inserted downstream an endogenous constitutive promoter [14]. Such chromosomal insertions led to BLG yields reaching ~2 μg/L of culture. In the present work, we adapted lactococcal tools to improve BLG production in *L. casei*. For this purpose, we tested different expression cassettes coding for BLG in fusion with a carrier protein and/or with a secretion-enhancer propeptide [15]. We quantified and analyzed the structure of the recombinant BLG, using two immunoassays, one specific for BLG in its native conformation and the other specific for reduced and carboxymethylated, i.e. denatured, BLG [16]. We thus succeeded to increase BLG production in *L. casei *BL23, up to 1 mg/L of culture. As the two-plasmid NICE system appeared to be leaky in *L. casei *BL23, we also integrated the *nisRK *genes (necessary for the nisin-inducible expression of BLG) into the bacterial chromosome. This led to a 1.5-fold increase in BLG production. Finally, we obtained higher yields of soluble BLG by using different conditions of nisin-induction.

## Results and Discussion

### Nuc is efficiently secreted by *L. casei*

**Figure 1:**
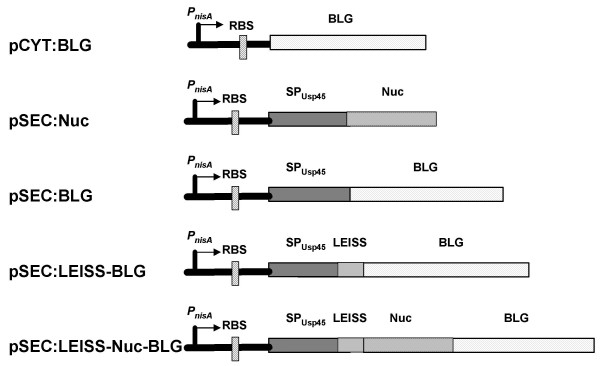
**Expression cassettes for production and secretion of staphylococcal nuclease and/or bovine β-lactoglobulin**. Nuc, staphylococcal nuclease, BLG, bovine β-lactoglobulin, P_*nisA*_, nisin-inducible lactococcal promoter; RBS, ribosome binding site of *usp45 *gene; SP_Usp45_, signal peptide of Usp45, LEISS, secretion-enhancer propeptide LEISSTCDA.

We first investigated the ability of the BL23 strain to produce and secrete the reporter staphylococcal nuclease (Nuc, [15]) under the transcriptional control of the nisin-inducible promoter P_*nisA*_. Two plasmids were used (Table 1). The plasmid pNZ9520 encodes the *nisRK *genes that are essential for the regulation of the *P*_*nisA *_promoter [17] and pSEC:Nuc contains a Nuc expression cassette with the *P*_*nisA *_promoter, the ribosome binding site (RBS_Usp45_) and the signal peptide (SP_Usp45_) of the major lactococcal secreted protein Usp45 ([18], Fig. 1).

**Table 1:** Bacterial strains and plasmids

Strain or plasmid	Characteristics	Reference
*L. casei*		
BL23	*L. casei *ATCC 393 (pLZ15^-^)	[26]
BL23(*int:nisRK*)	BL23 containing the *nisRK *genes integrated to the tRNA^Ser ^locus; obtained by transformation with pMEC10	This work
Plasmids		
pVE3655	Cm^r^, ori(pWV01), carries the nisin-inducible promoter P_*nisA*_	
pCYT:BLG	Cm^r^, ori(pWV01), with a DNA fragment encoding the BLG mature moiety expressed under P_nisA _transcriptional control	[6]
pSEC:BLG	Cm^r^, ori(pWV01), with a DNA fragment encoding the precursor SP_Usp45_-BLG expressed under P_nisA _transcriptional control	[6]
pSEC:LEISS-BLG	Cm^r^, ori(pWV01), with a DNA fragment encoding the precursor SP_Usp45_-LEISS-BLG expressed under P_nisA _transcriptional control	[8]
pSEC:LEISS-Nuc-BLG	Cm^r^, ori(pWV01), with a DNA fragment encoding the precursor SP_Usp45_-LEISS-Nuc-BLG expressed under P_nisA _transcriptional control	[8]
pSEC:Nuc	Cm^r^, ori(pWV01), with a DNA fragment encoding the precursor SP_Usp45_-Nuc expressed under under P_nisA _transcriptional control	[18]
pNZ9520	Em^r^, *nisRK *cloned in pIL253	[17]
pMEC10	Em^r^, integration plasmid containing the *nisRK *genes and the *int-attP *cassette for integration to the tRNA^Ser ^locus.	[21]

**Figure 2:**
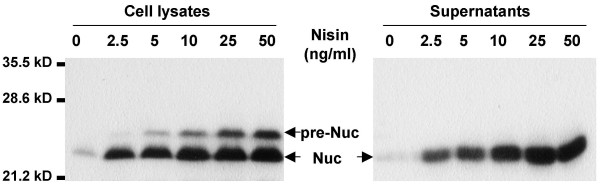
**Dose-dependent nisin induction of staphylococcal nuclease production and secretion**. *L. casei *BL23 strain was co-transformed with pSEC:Nuc and pNZ9520 plasmids. Protein extracts were analyzed by immunoblotting using rabbit polyclonal anti-Nuc antibodies as described in the Materials and Methods section. Arrows indicate positions of Nuc and its precursor form (pre-Nuc).

Nuc expression was induced with increasing concentrations of nisin up to 50 ng/mL (Fig. 2). Western blot analysis of protein extracts from cell lysates and supernatant fractions showed a dose-response correlation between nisin concentration and Nuc synthesis. The highest yield was obtained with 50 ng nisin/mL. We also detected an increasing release of mature Nuc into the culture medium in response to nisin induction, showing that SP_Usp45 _is functionally recognized and processed by the secretion machinery of the *L. casei *BL23 strain. As observed on Fig. 2, the efficient processing of the Nuc precursor is confirmed by the small amounts of SP_usp45_-Nuc found in cell lysates.

### Improved production and secretion of BLG in *L. casei*

We further investigated BLG production in *L. casei *with a construct (on pCYT plasmid, Table 1) targeting intracellular location and constructs (on pSEC plasmids, Table 1) encoding different fusion proteins previously designed for secretion of heterologous proteins in *L. lactis *[8].

#### Use of a lactococcal signal peptide

Intracellular production of BLG obtained with the pCYT:BLG plasmid was relatively low (78 μg/L of culture, Table 2). The small amount of BLG occasionally detected in the S fraction was certainly due to BLG release in the culture medium from lysed cells. Considering that secretion is an efficient way to increase the overall heterologous protein production in *L. lactis *[15], we tested whether enhancement of BLG secretion could improve BLG yields in *L. casei*. As SP_Usp45_-Nuc is efficiently secreted (Fig. 2), we first investigated the secretion of the precursor SP_Usp45_-BLG. Surprisingly, no secreted BLG was detected in the S fraction of *L. casei *BL23(pSEC:BLG) (Table 2). In contrast, we measured a significant enhancement of the insoluble intracellular BLG concentration (around 706 μg/L). Such effect of SP_Usp45 _has been already observed for production of heterologous proteins in *L. lactis *[6,19]. We suggest that presence of SP_Usp45 _sequence improved the translation of the *blg *gene but that the resulting protein was aggregating because of an inefficient recognition of the hybrid precursor by the secretion machinery. In *L. casei*, we thus found 92% of the recombinant BLG in the Ci fraction. Therefore, BLG in the Cs fraction represented only 8% of the total BLG production and only 1% of the soluble BLG exhibited a native conformation.

**Table 2:** Quantitative assays of BLG in different fractions of recombinant *L. casei *strains

Fraction^b^	Concentration of BLG (μg/L)^a^					
	
	BL23 + pNZ9520^c^					BL23(*int:nisRK*)^d^
		
	pSEC-Nuc	pCYT:BLG	pSEC:BLG	pSEC:LEISS-BLG	pSEC:LEISS-Nuc-BLG	pSEC:LEISS-Nuc-BLG
S (*% BLGn*)^e^	nd^f^	1.4 ± 2.2 (*43%*)	1.4 ± 1.8 (*0%*)	8.7 ± 2.3 (*40%*)	27 ± 3.7 (*46%*)	29.4 ± 6.4 (*46%*)
Cs (*% BLGn*)	nd	57.6 ± 10.3 (*27%*)	57.6 ± 27.3 (*1%*)	38.4 ± 12 (*1%*)	44.1 ± 12 (*8%*)	57.7 ± 6.3 (*8%*)
Ci	nd	19.2 ± 2.6	647.4 ± 301.1	975 ± 238.1	342.9 ± 128.7	526.9 ± 131.4
Total	nd	78.2 ± 6.5	706.4 ± 303.9	1022.1 ± 327.5	413.9 ± 116	613.9 ± 144.1

^a ^Values represent the means ± SD of at least three independent experiments; ^b ^S, supernatant; Cs, soluble cytoplasmic fraction; Ci, insoluble cytoplasmic fraction; ^c ^*L. casei *BL23 cells transformed with pNZ9520 plasmid; ^d ^*L. casei *BL23 cells with *nisRK *genes integrated in the chromosome; ^e ^Percentage of BLG in its native form; ^f ^nd, not detectable.

#### Use of a propeptide enhancer

The propeptide LEISSTCDA (LEISS) was inserted between the SP_Usp45 _and the mature moiety of the BLG. This insertion has been shown to improve secretion of heterologous proteins produced in *L. lactis *because of the presence of negatively charged residues [20]. For example, fusion of LEISS to BLG led to a 5-fold increase in BLG secretion in *L. lactis *[8]. In *L. casei*, this modification resulted similarly in a 6-fold increase in BLG secretion, reaching 9 μg/mL of culture. Total BLG production also increased from 706 μg/L to 1022 μg/L of culture, but mostly in the Ci fraction. As observed with SP_Usp45_-BLG in the Cs fraction, only 1% of soluble LEISS:BLG protein exhibited a native BLG conformation. However, the proportion of native BLG was significantly higher in the supernatant than in the cytoplasm since 40% of the BLG protein secreted in the culture medium displayed a native conformation.

#### Use of a carrier protein

**Figure 3:**
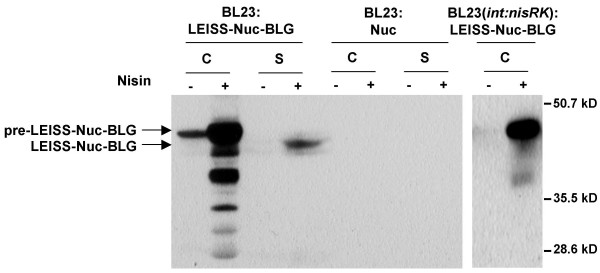
**LEISS-Nuc-BLG production and secretion by *L. casei *BL23 and BL23 (*int:nisRK*) strains**. Protein extracts were analyzed by immunoblotting using a specific anti-BLG monoclonal antibody for BL23 samples and rabbit polyclonal anti-Nuc antibodies for BL23 (*int:nisRK*) samples, as described in the Materials and Methods section. Arrows indicate the positions of LEISS-Nuc-BLG and precursor form.

Finally, we evaluated the effect of Nuc as carrier protein. Nuc protein was shown to improve immunogenicity of BLG and of a major BLG epitope after oral or subcutaneous administrations to mice[7,9]. Moreover, in *L. lactis*, insertion of Nuc between LEISS and the mature moiety of BLG resulted in a 2-fold increase in total BLG production but in a 4-fold decrease in secretion when compared with LEISS-BLG [8]. In contrast, Nuc insertion between LEISS and BLG led to a 3-fold higher secretion in *L. casei*. Secretion efficiency was evaluated to 6.5% of the total BLG production. As shown on the corresponding Western blot analysis (Fig. 3), only the mature form of LEISS-Nuc-BLG protein was secreted. Moreover, 46% of LEISS-Nuc-BLG protein exhibited a native conformation, showing thus that presence of Nuc does not influence the BLG folding since similar proportion of the native form was measured with LEISS-BLG. In the Cs fraction, proportion of the native form was even improved compared to LEISS-BLG since 8% of the fusion protein exhibited the BLG native form. Nevertheless, most of the LEISS-Nuc-BLG synthesized was located in the Ci fraction and intracellular LEISS-Nuc-BLG concentration was about 2.5-fold lower than intracellular LEISS-BLG concentration. As revealed by Western blot analysis (Fig. 3), accumulation of precursor SP_Usp45_-LEISS-Nuc-BLG in cell lysates resulted also in the presence of signals of lower molecular mass, certainly as a consequence of proteolytic degradation of misfolded recombinant protein.

Taken together, modifications used to improve BLG production in *L. lactis *are also effective in *L. casei*. Although BLG production in *L. casei *remains 2 to 10-fold lower than that obtained in *L. lactis *[8], we succeeded to obtain a substantial increase in BLG production and secretion. In both *L. casei *and *L. lactis*, the highest yield was obtained with the LEISS-Nuc-BLG form. While in *L. lactis *the optimal secretion was obtained with the LEISS-BLG form, the highest level of secretion in *L. casei *was reached with the LEISS-Nuc-BLG construct. Use of the two-plasmid NICE system resulted also in a 500-fold increase in BLG production compared to the level obtained with recombinant *L. casei *strains possessing one copy of the *blg *gene into their chromosome [14]. However, Western blot analysis of Nuc production (Fig. 2, lane 0) or LEISS-Nuc-BLG production (Fig. 3, first lane) revealed that the NICE system is relatively leaky in the absence of nisin induction. BLG production was then further optimized by investigating the conditions of nisin induction.

### Integration of *nisRK *genes into the *L. casei *chromosome

It has already been observed that the two-plasmid NICE system is less tightly regulated in *Lactobacillus *strains than in *L. lactis *strains [21,22]. In order to strengthen the regulation and to prevent the toxicity due to high expression levels of the nisin regulatory genes, the *nisRK *genes were integrated into the BL23 chromosome, as described by Pavan *et al *([21], see Materials and Methods). Competent cells of the resulting strain, BL23(*int*:*nisRK*), were transformed with the pSEC:LEISS-Nuc-BLG and intracellular BLG productions with or without nisin induction were analyzed by Western blot. As observed on Fig. 3, no LEISS-Nuc-BLG synthesis was detected in the absence of nisin, suggesting an improved regulation of the NICE system. Compared to BL23 co-transformed with pSEC:LEISS-Nuc-BLG and pNZ9520 plasmids, a 50% increase in BLG concentration was measured in the Ci fraction but neither concentration of soluble LEISS-Nuc-BLG, nor proportion of the native form in S and Cs fractions, were significantly improved (Table 2).

### Improved production of soluble BLG in *L. casei*

Recombinant *L. casei *are currently tested as delivery vehicle of BLG to the gastrointestinal tract of mice. Such experiments require high amounts of lactobacilli. Considering that addition of nisin at early growth phase resulted in a significant growth inhibition, different protocols allowing overnight production of bacterial biomass and nisin induction of *L. casei *cultures at higher cell density were tested. Consequently, bacterial pellets from overnight cultures were resuspended in fresh medium in order to remove lactic acid and other bacterial metabolites (protocol B, see Materials and Methods). After 1 h 30 at 37°C to restart bacterial growth, nisin induction was performed and maintained for 2 h at 37°C.

Interestingly, 74% of intracellular BLG obtained with protocol B remained soluble when only 10% of intracellular BLG obtained with the previously used protocol A were located in the Cs fraction (Table 3). Although total BLG yield with protocol B was nearly 2-fold lower, soluble BLG concentration was about 4-fold higher than that obtained with protocol A. The higher proportion of soluble BLG was certainly due to a lower growth rate at the time of induction. As already observed in *E. coli *[23-25], the folding capacity of the cell could become limiting at high expression rates. A slower rate of heterologous gene expression could then reduce inclusion bodies formation. However, it should be noted that, although proportion of soluble BLG is drastically enhanced, less than 1% of soluble BLG was detected in its native conformation. Since administration of *L. lactis *producing almost exclusively denatured BLG could partially prevent mice from sensitization [6,9], conformation of the recombinant BLG seemed not to be critical to induce a protective immune response. On the other hand, considering the processing of lactobacilli and their intracellular content by the host immune system, higher concentrations of soluble antigen could be advantageous for the development of a specific immune response by favoring antigen presentation to the mucosal immune system.

**Table 3:** Quantitative assays of BLG in different fractions of BL23(*int:nisRK*):LEISS-Nuc-BLG

Fraction^b^	Concentration (ng BLG/mg total protein)^a^	
	
	Protocol A^c^	Protocol B^c^
	
Cs (*% BLGn*)^d^	725,8 ± 33,7 (*8%*)	2481,6 ± 407,2 (*1%*)
Ci	6573,6 ± 631,6	884,7 ± 283,4
Total	7299.4 ± 597.9	3366.8 ± 356.4

^a ^Values represent the means ± SD of at least two independent experiments; ^b ^Cs, soluble cytoplasmic fraction; Ci, insoluble cytoplasmic fraction; ^c ^see nisin induction in Materials and Methods; ^d ^Percentage of BLG in its native form.

## Conclusion

We observed that fusion of the LEISS propeptide and Nuc, as initially described in *L. lactis*, improved both BLG production and secretion in *L. casei*. Furthermore, integration of *nisRK *genes into the *L. casei *BL23 chromosome allowed to strengthen nisin-dependent production of BLG and led to higher yields of recombinant protein. As previously mentioned in *L. lactis*, all these modifications did not improve the proportion of soluble BLG in the intracellular fraction [8]. Production of soluble BLG was nevertheless improved by performing nisin induction on *L. casei *cultures at higher cell density.

These recombinant strains were primarily designed to evaluate the potential advantages of using probiotic lactobacilli for the mucosal delivery of an antigen in a mouse model of allergy. This raised different questions such as whether we need *in situ *production and secretion of the antigen to induce or modulate an immune response or what is the most effective way of administration. In this regard, we are currently testing the BLG-producing *L. casei *for prophylactic and therapeutic treatments on mice, via delivery of recombinant lactobacilli by oral and intranasal administrations.

## Methods

### Bacterial strains and culture conditions

The bacterial strains used in this study are listed in Table 1. *L. casei *strains, derived from strain BL23 (ATCC 393 cured of plasmid pLZ15, [26]) were grown at 37°C in De Man-Rogosa-Sharpe broth ([27], Difco, BD, Le Pont de Claix, France). Plates were incubated in anaerobic jars for 2 days at 37°C in an Anaerocult A system (Merck). When required, erythromycin (Merck, Darmstadt, Germany) was added to the media at 5 μg/mL to select *L. casei *transformants. For promoter induction, nisin (Sigma, St Louis, MO, USA) was added at the required concentration (2.5–50 ng/mL).

### DNA manipulations

Purification of genomic DNA from *L. casei *was performed using the NucleoSpin Tissue kit (Macherey-Nagel, Hoerdt, France). Taq DNA polymerase was purchased from TakaraBio, Inc. (Otsu Shiga, Japan).

### Plasmids and strain constructs

The plasmids used in this study are listed in Table 1. *L. casei *was transformed by electroporation using a gene-pulser apparatus (Bio-rad Laboratories, Richmond, California) as previously described [28]. The pMEC10 plasmid was kindly provided by Dr. P. Hols. This plasmid, unable to replicate in *L. casei*, was introduced in electro-competent cells to integrate the *nisRK *genes into the genome of the BL23 strain [21]. Erythromycin-resistant integrants were analyzed by PCR and tested for functional nisin-induction. One of these clones, designated as BL23(*int:nisRK*), was further studied.

### Nisin induction

Two protocols were used in this study. Protocol A was performed for most experiments as follows: an overnight culture of *L. casei *BL23 was used to inoculate fresh medium at an initial OD_600 _of 0.35. After 1 h 30 at 37°C, nisin was added at the required concentration and strains were grown for 4–5 h at 37°C until an approximate OD_600 _of 1. Considering growth properties of the recombinant strains, we observed no significant difference in growth rates. *L. casei *cultures were thus harvested at similar exponential growth phase. For protocol B, cells grown to stationary phase by overnight culture were centrifuged at 8,000 × g for 5 min at 20°C and resuspended in 2.5 volumes of fresh medium. Optical density at 600 nm of *L. casei *cultures after resuspension in fresh medium was around 2.5. After 1 h 30 at 37°C in order to reinitiate bacterial growth, strains were induced with 25 ng nisin/mL for 2 h at 37°C. Optical density at 600 nm of *L. casei *cultures was then around 5.

### Western blot analysis

After nisin induction (as described above), *L. casei *cultures (2 mL) were pelleted by centrifugation at 8,000 × g for 5 min at 4°C. Secreted proteins in the supernatant (1.7 mL) were precipitated with trichloroacetic acid (15% final concentration). After centrifugation at 18,000 × g for 30 min at 4°C, the resulting pellet was resuspended in 25 μL of 50 mM NaOH and 25 μL of 2× Laemmli buffer. The cell pellet was washed once and resuspended in 100 μL of 50 mM Tris-HCl pH 7.5, 5 mM EDTA. Cells were disrupted with glass beads and 100 μL of 2× Laemmli buffer (supplemented with 5% β-mercaptoethanol as reducing agent) were added. Protein sample concentration was adjusted to the cell density of the producing culture if needed. Samples were loaded for SDS-PAGE analysis followed by Western blotting using polyclonal anti-Nuc antibodies or specific anti-BLG monoclonal antibody Blg-92R [16].

### Immunoassays

After nisin induction (as described above), cells were pelleted by centrifugation at 8,000 × g for 5 min at 4°C and the supernatant (S) was collected. Cells were resuspended in 50 mM Tris-HCl, pH7.5, 5 mM EDTA and cell density was normalized according to OD_600_. The soluble cytoplasmic protein (Cs) was extracted by disrupting cells with glass beads. After centrifugation (15,000 × g, 15 min, 4°C), the supernatant corresponding to the Cs fraction was collected. The pellet was resuspended in 50 mM Tris-HCl, pH7.5, 8 M urea, 100 mM dithiothreitol, for 1 h at room temperature in order to solubilize BLG included in aggregates. After centrifugation (15,000 × g, 15 min, 4°C), the supernatant containing the resolubilized insoluble cytoplasmic protein (Ci) was collected and dialyzed against 20 mM Tris-HCl, pH 7.5. Amounts of the BLG native (BLGn) and denatured (BLGd) forms in the S and Cs fractions, and of resolubilized BLG from the Ci fraction, were determined by immunoassays based on the use of pairs of monoclonal antibodies specific to either the native form or the reduced and carboxymethylated, i.e. denatured, form of BLG [16]. Briefly, 96-well microtiter plates (Maxisorp; Nunc, Roskilde, Denmark) were coated with a first monoclonal antibody (mAb, capture antibody) specific for BLGn or BLGd. Then, 50 μL of sample and 50 μL of tracer (second mAb labelled with acetylcholinesterase) were added. Native BLG and reduced and carboxymethylated BLG were used as standards for quantification of BLGn and BLGd, respectively. After 18 h of incubation at 4°C, the plates were extensively washed, and solid-phase-bound AChE activity was measured by the method of Ellman [29]. Intracellular BLG concentration was also calculated with respect to the total protein determined by the BCA protein assay (Pierce Biotechnology, Rockford, IL 61105, USA).

## Competing interests

The author(s) declare that they have no competing interests.
